# Erratum to: *Education:* The role of the first siderophore cephalosporin Fetcroja^®^ (cefiderocol) in UK clinical practice: introduction; Antibiotic resistance during and beyond COVID-19; *Education:* An introduction to the core data for cefiderocol with reflections for a possible role within UK clinical practice; *Education:* A compassionate use of cefiderocol to treat osteomyelitis caused by an XDR *Pseudomonas aeruginosa*; Compassionate use of cefiderocol for carbapenem-resistant *Acinetobacter baumannii* prosthetic joint infection; *Education:* An overview from the author of ‘Cefiderocol as rescue therapy for *Acinetobacter baumannii* and other carbapenem-resistant Gram-negative infections in intensive care unit patients’

**DOI:** 10.1093/jacamr/dlab109

**Published:** 2021-08-28

*JAC-Antimicrobial Resistance* 2021; https://doi.org/10.1093/jacamr/dlab051

*JAC-Antimicrobial Resistance* 2021; https://doi.org/10.1093/jacamr/dlab052

*JAC-Antimicrobial Resistance* 2021; https://doi.org/10.1093/jacamr/dlab053

*JAC-Antimicrobial Resistance* 2021; https://doi.org/10.1093/jacamr/dlab054

*JAC-Antimicrobial Resistance* 2021; https://doi.org/10.1093/jacamr/dlab055

*JAC-Antimicrobial Resistance* 2021; https://doi.org/10.1093/jacamr/dlab056

In the original published versions of these articles, the link to the prescribing information for cefiderocol was missing. This has now been corrected in the published versions of the articles. The authors apologize for this error.

